# Wip1 and ATM in tumor evolution: role for BRCA1

**DOI:** 10.18632/oncotarget.1661

**Published:** 2013-12-09

**Authors:** Doria Filipponi, Dmitry V. Bulavin

**Affiliations:** Institute of Molecular and Cell Biology, Proteos, Singapore; Institute of Molecular and Cell Biology, Proteos, Singapore

Sequential accumulation of mutations is a central event that drives the conversion of normal cells into cancer. The recent advent of whole-genome sequencing data highlighted the existence of several hundred to thousand's of mutations in tumors affecting the integrity of the genome. In order to maintain genomic stability, and thus to suppress tumorigenesis, eukaryotic cells have evolved several defense mechanisms to prevent accumulation of cells carrying oncogenic mutations. Among those, the ataxia telangiectasia mutated (ATM) is one of the master regulators of DNA damage-induced signaling that has also been implicated in the regulation of an oncogene-induced stress. ATM mutations have been recently associated with cancer predisposition further highlighting its role as a potent tumor suppressor. In line with these observations, we recently found that ATM re-activation by manipulating WIP1 levels could potentially decrease the rate of tumor evolution (Filipponi D et al., Cancer Cell *2013; 24,* 528-541).

WIP1, a serine/threonine phosphatase encoded by the PPM1D gene, is overexpressed, amplified and mutated in several types of cancer including those of the mammary gland and colon. WIP1 plays a critical role in accelerating tumorigenesis by inactivating p53 and INK4a/ARF tumor suppressor pathways. In addition, WIP1 is involved in cancer progression through regulation of several DNA repair pathways including base excision repair (BER) as well as those controlled by ATM.

By analyzing germ cells in WIP1 knockout mice we observed heterochromatin silencing that was associated with increased DNA methylation. Since heterochromatin-associated sequences are aberrantly re-expressed in cancer contributing to genome stability, this prompted us to investigate the impact of WIP1 deletions in cancer cells. We found a substantially reduced expression of repetitive DNA sequences which correlated with increased DNA methylation. WIP1 regulates the ATM signaling pathway and we found increased phosphorylation levels of BRCA1, a downstream target of ATM. Through phosphorylation of BRCA1 (p-BRCA1), ATM signaling established the patterns of DNA methylation at CG-rich DNA sequences to silence heterochromatin regions in WIP1 deleted cells. Chromatin immunoprecipitation experiments indicated that following WIP1 deletion p-BRCA1 occupied those regions and functioned downstream of ATM to silencing CG-rich DNA sequences. In this model, BRCA1 directs the targeting of the HP1-DNMT3B complex to heterochromatic sequences, ultimately establishing de-novo patterns of DNA methylation while silencing heterochromatin-associated sequences including retrotransposons.

WIP1 played a critical role in modulating DNA methylation patterns but also orchestrated the targeting of Activation-Induced cytidine Deaminase (AID) to CG-rich sequences. AID is a central enzyme for both class switch recombination and formation of somatic hypermutations. AID generates U:G mismatch that may trigger A/T and C/G pair mutations if not correctly repaired by BER. In turn, WIP1 overexpression resulted in increased H3K4me3 at CG-rich regions most likely creating a platform for AID recruitment that could potentially facilitate the generation of an U:G mismatch. Since WIP1 also blocks BER pathway activity, this mismatch may not be properly repaired thus increasing the mutation frequency.

An accumulation of mutations and genomic instability play detrimental roles in cancer progression however the molecular mechanisms explaining their appearance are not fully understood. This raised the question as to whether WIP1-dependent modulation of CG-rich sequences and enhanced recruitment of AID could play a role in tumorigenesis. By analyzing somatic mutation datasets for human breast cancers we found a strong positive correlation between the occurrence of C-to-T transitions and total mutation load with both WIP1 copy numbers and WIP1 expression levels. It is important to understand whether other genetic alterations including deletions, insertions, duplications and chromosome translocations are regulated through WIP1-BRCA1-AID pathway. Despite several remaining questions, our initial analysis suggests the ability of WIP1 to de-repress heterochromatin-associated sequences including retroelements. Thus, WIP1 overexpression in human cancers could be one of the mechanisms contributing to genomic instability and cancer evolution.

